# Modeling suggests fossil fuel emissions have been driving increased land carbon uptake since the turn of the 20th Century

**DOI:** 10.1038/s41598-020-66103-9

**Published:** 2020-06-03

**Authors:** Christopher R. Schwalm, Deborah N. Huntinzger, Anna M. Michalak, Kevin Schaefer, Joshua B. Fisher, Yuanyuan Fang, Yaxing Wei

**Affiliations:** 1 Woods Hole Research Center, Falmouth, MA 02540 USA; 2 Center for Ecosystem Science and Society, Northern Arizona University, Flagstaff, AZ 86011 USA; 3 School of Earth & Sustainability, Northern Arizona University, Flagstaff, AZ 86011 USA; 4 Department of Global Ecology, Carnegie Institution for Science, Stanford, CA 94305 USA; 5 National Snow and Ice Data Center, Boulder, CO 80309 USA; 6 Jet Propulsion Laboratory, California Institute of Technology, 4800 Oak Grove Dr., Pasadena, CA 91109 USA; 7 Environmental Sciences Division, Oak Ridge National Laboratory, Oak Ridge, Tennessee 37831 USA

**Keywords:** Biogeochemistry, Climate sciences, Ecology, Environmental sciences

## Abstract

Terrestrial vegetation removes CO_2_ from the atmosphere; an important climate regulation service that slows global warming. This 119 Pg C per annum transfer of CO_2_ into plants—gross primary productivity (GPP)—is the largest land carbon flux globally. While understanding past and anticipated future GPP changes is necessary to support carbon management, the factors driving long-term changes in GPP are largely unknown. Here we show that 1901 to 2010 changes in GPP have been dominated by anthropogenic activity. Our dual constraint attribution approach provides three insights into the spatiotemporal patterns of GPP change. First, anthropogenic controls on GPP change have increased from 57% (1901 decade) to 94% (2001 decade) of the vegetated land surface. Second, CO_2_ fertilization and nitro  gen deposition are the most important drivers of change, 19.8 and 11.1 Pg C per annum (2001 decade) respectively, especially in the tropics and industrialized areas since the 1970’s. Third, changes in climate have functioned as fertilization to enhance GPP (1.4 Pg C per annum in the 2001 decade). These findings suggest that, from a land carbon balance perspective, the Anthropocene began over 100 years ago and that global change drivers have allowed GPP uptake to keep pace with anthropogenic emissions.

## Introduction

Our understanding of changes in Earth system processes depends on models^1,2^. Model-based attribution, quantifying the importance and magnitude of causal factors for a detected change^3^, is routinely used to assess the relative contributions of anthropogenic factors and natural variability on Earth system phenomena, ranging from precipitation extremes to decadal-scale changes in net carbon uptake^4–8^. For land carbon metabolism, attribution typically focuses on net land uptake of CO_2_ (ref. ^9^). While this quantity is important to inform climate policy, e.g., Paris Climate Accords, it is the disequilibrium across many processes—such as heterotrophic respiration and fire emissions—with uncertain magnitude and spatiotemporal patterns^7,9,10^. This suggests that an improved understanding of the net land-atmosphere CO_2_ signal can be achieved by examining each component flux, and relevant drivers of change, individually.

Here we use a novel dual constraint approach to attribute centennial scale changes in GPP at grid cell to global scales. GPP is of central importance for the net carbon balance as it represents the entry of carbon into land ecosystems such that all other processes are downstream. We quantify changes in GPP due to natural climate variability, land use/land cover change, greenhouse gases, and nitrogen deposition. Our approach uses two broad ensembles of Earth system models (ESMs): (1) a 11-member ensemble of observation-driven land surface models—corresponding to the land component of ESMs in offline mode—from the Multi-scale synthesis and Terrestrial Model Intercomparison Project (MsTMIP) (ref. ^11^); and (2) an 13-member ensemble of fully coupled ESMs from CMIP5, the fifth phase of the Coupled Model Intercomparison Project^12^. We also use machine learning to recover the change in GPP for the individual offline climatic factors of heat (near-surface air temperature), water (precipitation), and light (downwelling shortwave radiation). This is based on the emulation (see Methods) of MsTMIP where only climate varies and then sequentially retrieving the contribution of each climatic factor by simulation differencing^9^.

**Figure 1:**
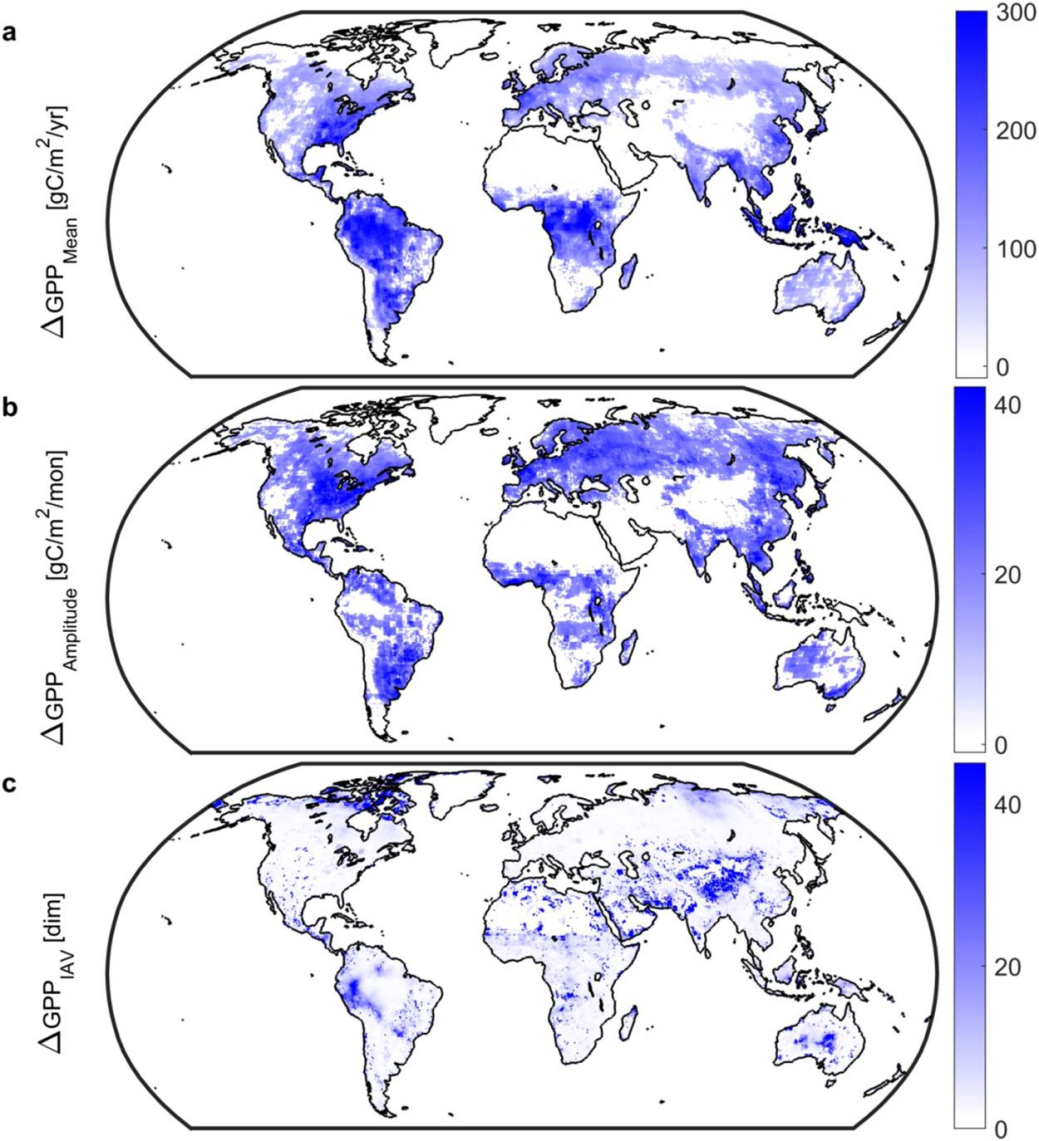
Long-term (1901–2010) changes in gross primary productivity (GPP). Changes in (**a**), mean annual GPP.; (**b**), monthly GPP amplitude.; and (**c)**, interannual variability. Mean and amplitude values calculated as the difference between 1981–2010 and 1901–1930 periods; positive values indicate an increase over time. Interannual variability, an index of volatility in land ecosystem carbon metabolism, is the ratio of standard deviations using deseasonalized GPP, e.g., a value of 2 indicates that 1981–2010 variability in gross uptake is twice that of the 1901–1930 period. All maps based on CMIP5 and MsTMIP. White grid cells are water, barren, or exhibited no significant change. Note difference in color scales. Figure created in Matlab version R2019a (http://www.mathworks.com/products/matlab/).

Enhanced GPP is the basis by which land ecosystems buffer climate change. Model-based reconstructions indicate significant changes in gross uptake of carbon by the vegetated land surface over the 20^th^ Century. From 1901 to 2010 GPP has increased globally (Fig. 1) by 10.5 Pg C per annum (uncertainty range: +8.2 to +12.4 Pg C per annum), in qualitative agreement with long-term atmospheric records of carbonyl sulfide^13^, and equivalent to 9% of current satellite-era global GPP of 119 Pg C per annum^2^. Increases in GPP are centered on the tropics as well as forested regions of the USA and Eurasia. In addition, both amplitude (difference between seasonal maximum and minimum) and volatility (changes in year-to-year variation) have increased. Changes in amplitude are most pronounced in the Northern Hemisphere (Fig. 1) and are overwhelmingly positive; by area 60% of vegetated land ecosystems have seen an increase versus only 2% where a decline occurred. The spatial footprint of changes in volatility shows that 62% of vegetated land ecosystems have seen at least a doubling of volatility versus <1% where volatility decreased. The largest changes in volatility, where year-to-year changes are at least tenfold larger in the satellite-era, are centered on the northern high latitudes and the Eurasian Steppe (Fig. 1), supporting the importance of these regions—particularly arid and dryland systems–in driving the interannual variability and trend of net carbon uptake^14^.

**Figure 2:**
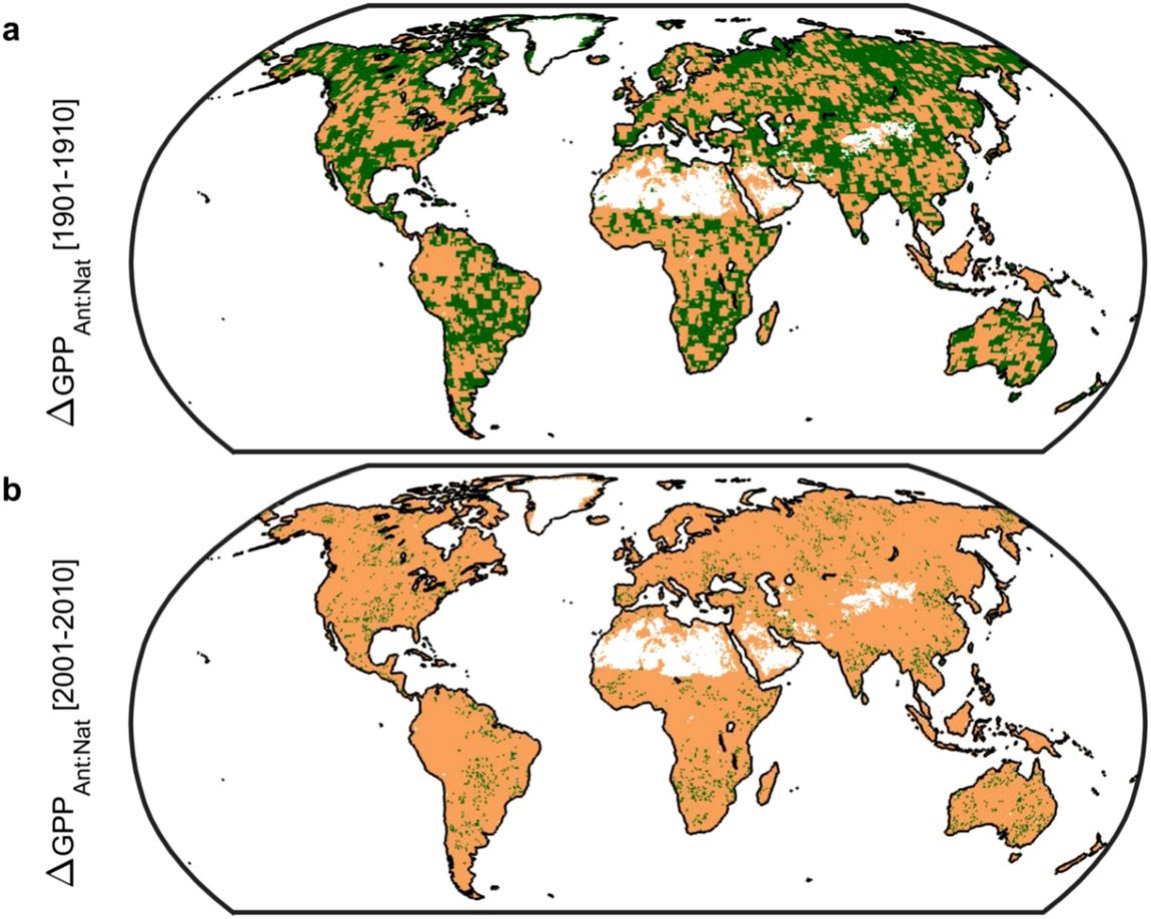
Spatial pattern of changes in gross primary productivity (GPP) due to anthropogenic forcings. (**a**), Map of 1901 decade. (**b**), Map of 2001 decade. Areal extent of GPP change due to anthropogenic forcings: 57% and 94% for 1901 and 2001 decades respectively. Values calculated as the ratio of absolute values of anthropogenic to natural forcing-induced changes in GPP using CMIP5 models only (see Methods). A ratio greater than unity (brown) indicates that anthropogenic forcings (primarily well-mixed greenhouse gases) dominate; whereas green indicates natural forcings (solar irradiance and volcanic aerosols) dominate. Figure created in Matlab version R2019a (http://www.mathworks.com/products/matlab/).

We find that anthropogenic forcings—primarily well-mixed greenhouse gases (see Methods)—are the source of these changes in GPP. Indeed, from the perspective of GPP we had already entered the Anthropocene, where human agency represents the most important geological forcing^15^, no later than the turn of the 20^th^ Century. Integrated globally, the impactof anthropogenic forcings has increased from -0.1 (1901 decade) to +15.6 Pg C per annum (2001 decade) and acts to enhance GPP. In contrast, natural forcings (solar irradiance and volcanic aerosols) show an ever changing, over space and time, pattern of small effect sizes (typically <1 Pg C per annum globally in absolute value with a mean of -0.02 Pg C per annum from 1901 to 2010; Extended Data Fig. 1) with the largest volatility in the tropics. Spatial footprints in the first decade of both the 20^th^ and 21^st^ Centuries show a predominance of anthropogenic factors (Fig. 2) with changes in GPP across 57% (1901 decade) and 94% (2001 decade) of all vegetated land ecosystems linked to human agency. Through time there is a clear trend toward total anthropogenic control of changes in GPP.

**Figure 3:**
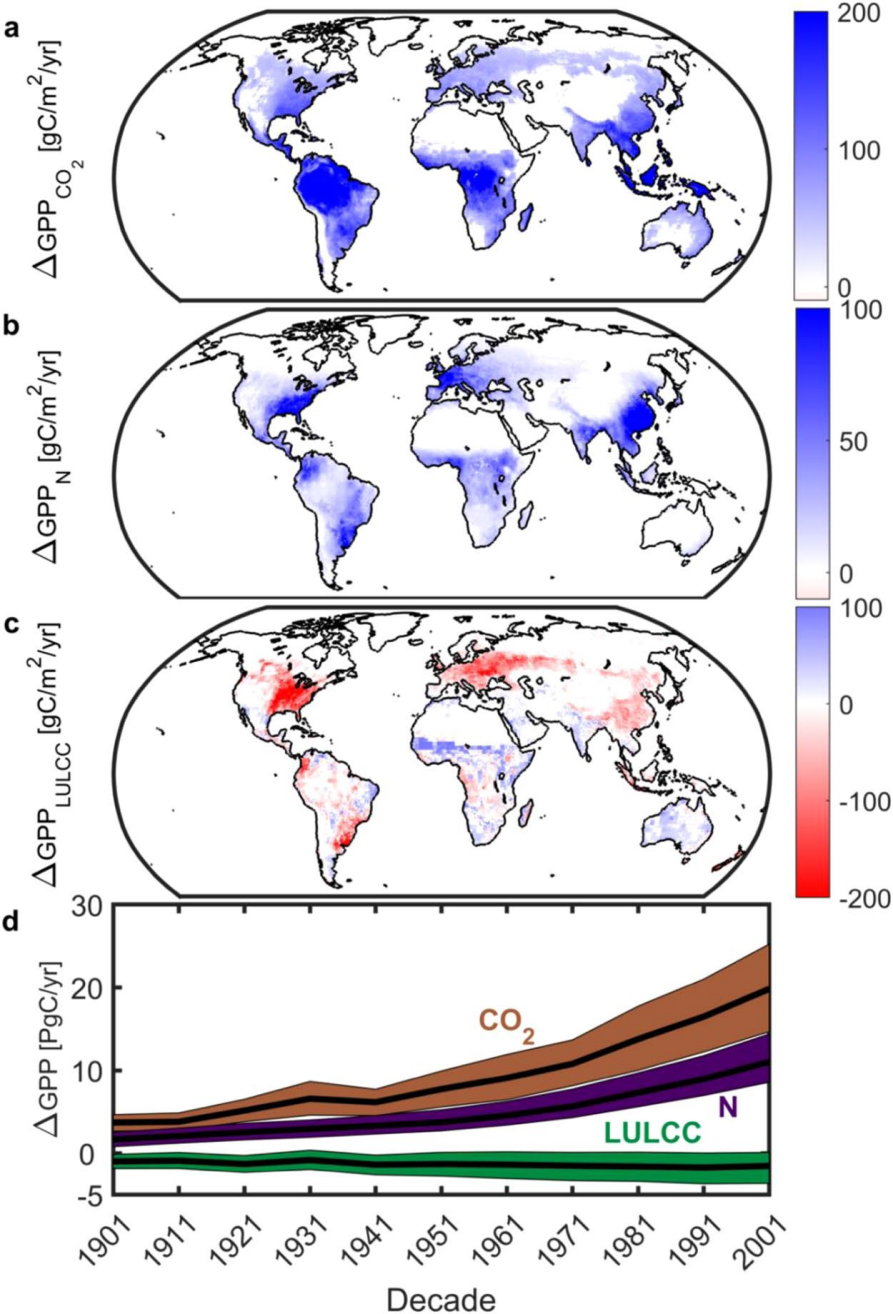
Anthropogenic controls of changes in gross primary productivity (GPP). Spatial long-term mean (1901–2010) changes in GPP due to (**a**), CO_2_ fertilization (CMIP5 and MsTMIP); (**b**), nitrogen deposition (MsTMIP only); and (**c**), LULCC (CMIP5 and MsTMIP). White grid cells are water, barren, or exhibited no significant change. Note difference in color scales. (**d**), Decadal changes in GPP due to CO_2_ fertilization (brown), nitrogen deposition (purple) and land use and land cover change (LULCC; green). Color envelope: 90% confidence interval around mean (black line). Figure created in Matlab version R2019a (http://www.mathworks.com/products/matlab/).

Among individual attribution factors considered, changes associated with the CO_2_ fertilization effect and nitrogen deposition are largest (Fig. 3). The CO_2_ effect has monotonically increased from +3.7 (1901 decade) to +19.8 Pg C per annum (2001 decade); a fivefold increase now equivalent to 17% of contemporary global GPP^2^. The impact of CO_2_ fertilization is most pronounced in the tropics and Southeast Asia (Fig. 3) and shows an increasing trend and spatial footprint poleward from the equator since the 1960’s (Extended Data Fig. 2). Nitrogen deposition (Fig. 3) has similarly acted to enhance GPP. This effect has grown from +1.6 (1901 decade) to +11.1 Pg C per annum (2001 decade) and is focused on industrialized areas in North America, Europe, and China (Extended Data Fig. 2). In contrast, land use and land cover change (LULCC) is associated with a negative impact globally (-1.3 Pg C per annum across all decades or 1% of contemporary global GPP) and the most relative uncertainty (Fig. 3). These impacts —driven by degrading high GPP systems (forests) to low GPP systems (shrublands)—are dominant in the eastern USA, Europe, China, and the tropics. Unlike CO_2_ and nitrogen deposition, the LULCC effect is less variable in time; it shows a relatively static spatial footprint through the 20^th^ Century (Extended Data Fig. 2).

**Figure 4:**
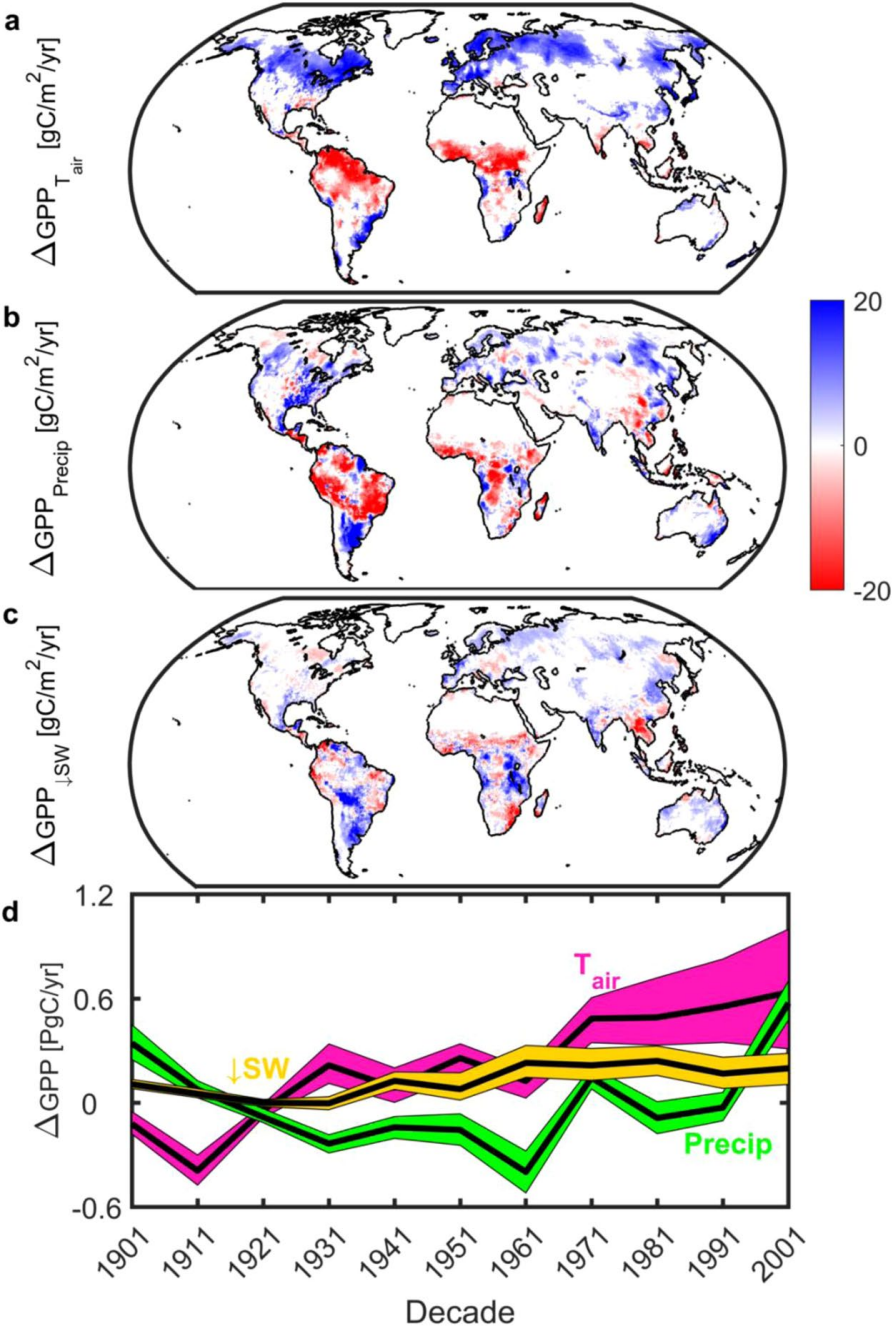
Climatic controls of changes in gross primary productivity (GPP). Spatial long-term mean (1901–2010) changes in GPP due to (**a**), air temperature; (**b**), precipitation; and (**c**), downwelling shortwave radiation. White grid cells are water, barren, or exhibited no significant change. (**d**), Decadal changes in GPP due to air temperature (cyan), precipitation (green) and downwelling shortwave radiation (gold). Color envelope: 90% confidence interval around mean (black line). All values derived from MsTMIP only. Figure created in Matlab version R2019a (http://www.mathworks.com/products/matlab/).

**Figure 5:**
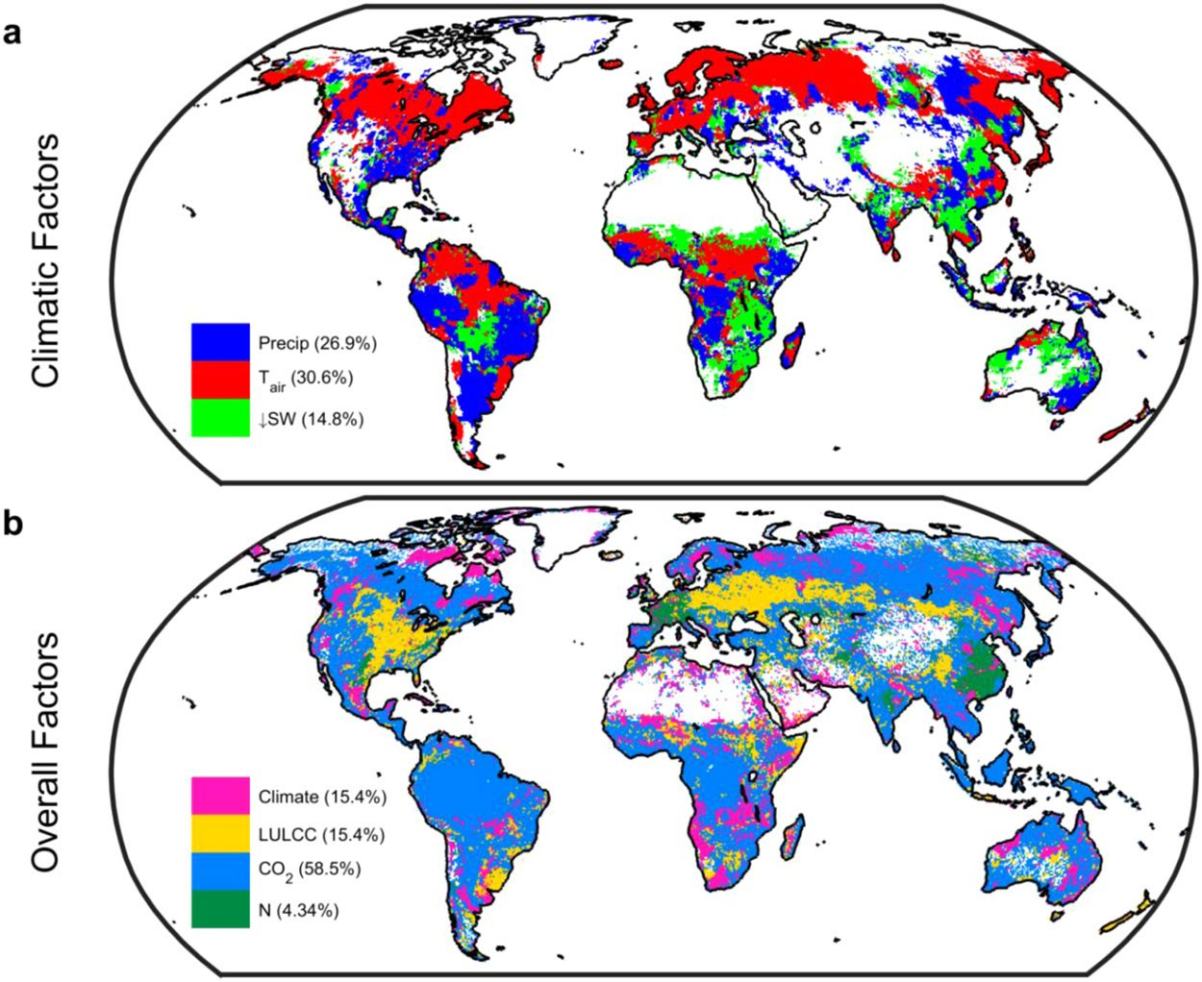
Controlling factors for changes in gross primary productivity (GPP). Spatial long-term mean (1901–2010) changes in GPP due to (**a**), individual climatic factors (emulator-based; see Methods) and; (**b**), climate, land use and land cover change (LULCC), CO_2_ fertilization, and nitrogen deposition (N) factors (based on simulation differencing). Values in parenthesis give percent of vegetated land surface where the effect predominates. Maps derived using MsTMIP models only (see Methods). White grid cells are water, barren, or exhibited no significant change (28% of the vegetated land surface for individual climatic factors and 6% for overall factors). Figure created in Matlab version R2019a (http://www.mathworks.com/products/matlab/).

Using a machine learning-based approach to decompose the net climate signal from the MsTMIP ensemble into temperature, precipitation, and radiation effects (see Methods) we find that climate change has functioned as climate fertilization—changes in climate have acted to increase gross uptake. Globally this effect is modest (Fig. 4), with a long-term average of +0.3 Pg C per annum, but has increased in recent decades to +1.4 Pg C per annum (2001 decade), equivalent to 50% of the contemporary net land sink^1^. Of all three climate effects temperature elicits the largest changes in GPP (Fig. 4) and has the largest spatial footprint (Fig. 5) while changes in radiation exhibit only a weak influence on GPP. Spatially, and in agreement with the effect of growing season temperature on peak GPP^16^, we find that temperature has enhanced GPP in the mid to northern latitudes. In contrast, 20^th^ Century changes in temperature and precipitation have acted to depress GPP in the tropics (Fig. 4). Even though changes in climate are important regionally and exhibit a similar overall areal extent as LULCC, the spatial footprint of changes in GPP is driven by CO_2_ fertilization. Across 58% of the vegetated land surface changes in CO_2_ serve as the most dominant factor (Fig. 5).

While ESMs agree that anthropogenic factors have dominated changes in GPP over the 20^th^ Century, care is warranted in interpretation. What constitutes an anthropogenic forcing varies across the CMIP5 ensemble and not all MsMTIP models simulate each sequential control. This is reflected in the confidence bounds (see Methods) for each factor. The total change due to CMIP5 anthropogenic forcings in the 2001 decade is +15.6 Pg C per annum, albeit with substantial uncertainty (+10.4 to +43.5 Pg C per annum). This is lower than but consistent with the corresponding figure from MsTMIP—equating the effects of CO_2_ fertilization, nitrogen deposition, and LULCC to anthropogenic forcings—of +25.6 Pg C per annum (uncertainty range: +16.1 to +35.8 Pg C per annum). This, in turn, matches the sum, +29.3 Pg C per annum (uncertainty range: +19.6 to +39.7 Pg C per annum), of individual anthropogenic factors (CO_2_ fertilization, nitrogen deposition, and LULCC) explicitly considered across both CMIP5 and MsTMIP ensembles. This consistency is supported by the similar effect sizes of CO_2_ fertilization and LULCC individually (nitrogen subsidy is only addressed in MsTMIP) across both ensembles (Extended Data Fig. 3). Finally, adjusting the CO_2_ effect downward to correct for the missing (and negative) vegetation-carbon feedback component of CO_2_ fertilization^17^ lowers this human agency effect to +24.8 Pg C per annum, again consistent with both CMIP5 and MsTMIP ensembles. In any case, the dominance of anthropogenic drivers in controlling changes in GPP since 1901 is clear, with CO_2_ fertilization and nitrogen subsidy the most important drivers in time, space (for CO_2_ only), and magnitude.

Despite this secular trend in GPP, there is substantial unresolved ambiguity surrounding both leading anthropogenic drivers in CMIP5 and MsTMIP. Evidence supporting the CO_2_ fertilization effect—the dominant driver of GPP enhancement since 1901—is supported by first principles of plant physiology but is otherwise mixed. According to recent studies, the CO_2_ fertilization effect is simultaneously underestimated for GPP in ESMs^18^, overestimated for NPP (GPP after autotrophic respiration has been subtracted) relative to satellite-derived products^19^, or not present in biomass growth for undisturbed tropical forests^20^ or in tree growth for western Canadian subalpine forests^21^. In contrast, a 100 ppm increase (roughly the 20^th^ Century) in CO_2_ is linked to a 35% increase in net photosynthesis based on isotopic signatures of deuterium in historic plant material and manipulation experiments^22^. Furthermore, when warming and CO_2_ fertilization are considered jointly, the latter is not associated with any growth enhancement, as seen in the Canadian boreal forest^23^ and in high-elevation forests of central Mexico^24^. Moreover, there are clear indications of sink saturation, suggesting on upper limit of uptake, especially in forest systems^25^. Finally, neither CMIP5 nor MsTMIP allow the vegetation-carbon feedback component of increasing CO_2_ concentrations to be quantified, potentially resulting in a 30% overestimation of the CO_2_ fertilization effect^17^.

A similar ambiguity concerns the effects of nitrogen subsidy. These occur in the context of a critical load past which additional nitrogen inputs have potentially deleterious impacts on ecosystem function, from mortality to changes in community composition, carbon allocation and growth rates^26,27^. For extratropical forests this critical load is estimated at 10 to 20 kg N/ha per annum overall^26^ but can be significantly lower for specific forest types, e.g., USA northern forests show declined survivorship at as low as 3 kg N/ha per annum^27^. In tropical forests the knowledge base is more limited and experimental evidence for a significant positive effect equivocal^28,29^. This holds especially in primary and lowland forests and is linked to phosphorous availability^28,30^, which is not addressed in either ensemble. Focusing on MsTMIP, where nitrogen subsidy increases by a factor of five, the percent area of impacted extratropical forests where positive nitrogen subsidy exceeds the minimum threshold increases monotonically from 1% (1901 decade) to 29% (2001 decade). External nitrogen inputs also always enhance gross uptake in tropical forests (Fig. 3), with a mean subsidy of 3.5 kg N/ha per annum—increasing monotonically from 1.1 (1901 decade) to 8.6 kg N/ha per annum (2001 decade)—eliciting a mean GPP increase of 30.1 g C/m^2^ per annum.

Both CMIP5 and MsTMIP heavily condition on the CO_2_ fertilization effect and—especially for MsTMIP models with carbon-nitrogen coupling^9^—external nitrogen to drive positive changes in gross carbon uptake. This occurs without any sign of agnostic effects or saturation; both nitrogen subsidy and levels of CO_2_ are highly correlated (ρ = 0.99) with GPP across decades globally. This is problematic in that resolving the efficacy of both anthropogenic drivers is a prerequisite to reducing the persistent and large uncertainties in the carbon cycle-climate feedback^10,31^. Beyond this, there are other innovations needed to narrow the range in simulated values. Especially relevant for changes in GPP is the treatment of agricultural systems where there is strong evidence that modeled estimates of GPP are systematically biased low^32^. As CMIP5 and MsTMIP do not include explicit crop modeling, attributed changes in GPP, particularly in the context of LULCC, are likely skewed. Evidence that recent enhanced global GPP is driven by croplands^33^ reinforces the nascent trend of embedding agriculture submodules into coupled Earth system models^32,34^.

More generally, state-of-the-art land modeling frameworks still lack processes that materially impact trajectories of GPP; such as nutrient cycling, dynamic vegetation and disturbance^35,36^. The increasing complexity of modeling frameworks and questions asked of them itself requires an updated approach to address heterogeneity in process representation, tiling schemes that vary by process within model, and tracking changes in parametrizable ecosystem properties^36^. Focusing on the lead anthropogenic drivers over the 20^th^ Century, knowledge base improvements for the CO_2_ fertilization effect and explicit parameterizable linkages between carbon cycling to nitrogen and phosphorus biogeochemistry^37^ are crucial to better constrain global GPP. This, in turn, points to the need of continuous iteration between modeling, data science, and experimental communities to improve predictive capacity^36^. As there is already evidence that future estimates of net carbon cycling may be biased upward when nutrient limitation is not considered^9,38^, a more robust treatment is more urgent still given the diminishing climate policy window to effect meaningful change in the trajectory of global change, past drivers of GPP enhancement notwithstanding.

## Methods

### Approach

We use historical reconstructions of gross primary productivity (GPP) from offline and fully coupled state-of-the-art Earth system models (ESMs). Offline reconstructions are drawn from 11 terrestrial biosphere simulators in the Multi-scale synthesis and Terrestrial Model Intercomparison Project (MsTMIP Version 1; 10.3334/ORNLDAAC/1225) (ref. ^11,39^). MsTMIP uses a constrained simulation protocol (driving data, vegetation cover, boundary conditions, and steady-state spin-up protocol are all standardized; only model structure varies). All runs use observation-based driving d ata to replicate 1901 to 2010 historical conditions^34^ with a semi-factorial design where time-varying factors of climate (SG1), land cover and land use change (LULCC) (SG2), [CO_2_] (SG3), and nitrogen deposition (BG1) are sequentially enabled after steady-state (RG1)—the reference run^11,39^ based on a repeated cycle of a randomized 30-yr block of trend-free weather—is achieved (MsTMIP simulation name in parenthesis). MsTMIP models used for this study are CLM, CLM4VIC, DLEM, GTEC, ISAM, LPJ-wsl, ORCHIDEE-LSCE, SiBCASA, TEM6, VEGAS2.1 and VISIT. Only CLM, CLM4VIC, DLEM and TEM6 simulate BG1, i.e., have carbon-nitrogen coupling.

Fully coupled reconstructions for 1901 to 2010 for 13 ESMs (Extended Data Table 1) are taken from the CMIP5 archive (http://cmip-pcmdi.llnl.gov/cmip5/data_portal.html). CMIP5, the fifth phase of the Coupled Model Intercomparison Project, serves as a central repository for ESM simulations that inform the Fifth Assessment Report (AR5) of the Intergovernmental Panel on Climate Change (IPCC) (https://ipcc.ch/report/ar5/). CMIP5 is designed to provide a multi-model framework to investigate model differences in carbon cycle and cloud-based feedbacks as well as climate predictability and the range in ESM responses from similar forcing inputs^12^. For this study ESMs are chosen based on simulation experiment availability. Each ESM has, at a minimum, a preindustrial control (piControl) and historical (historical) experiments (CMIP5 simulation experiment name in parenthesis; see Extended Data Table 1). In addition, experiments that differ from piControl or historical by a single factor (or factor set, e.g., all anthropogenic forcings) are also included: (1) with greenhouse gas forcing only (historicalGHG), (2) with natural forcing only (historicalNat), (3) with LULCC only and/or (4) with anthropogenic forcings only (variants of historicalMisc). Finally, two fully coupled carbon/climate experiments are included if available: (1) the carbon cycle sees preindustrial levels of CO_2_ but the radiation code sees historical conditions (esmFdbk2) and (2) the radiation code sees preindustrial levels of CO_2_ but the carbon cycle sees historical CO_2_ (esmFixClim2). In all cases only one realization per ESM is used; land carbon cycling is driven by model structure and therefore insensitive to initial conditions.

For both offline and coupled reconstructions we analyze annual values as global aggregates as well as by grid cell. For mapped values CMIP5 reconstructions are resampled to a half-degree spatial resolution to match the MsTMIP land mask and use the barren mask from a contemporary upscaled eddy covariance product^2^. Our results are based on integration over the full ensemble of ESMs—the consensus ensemble mean^40^—using “one-model-one-vote” (ref. ^41^) and assume that an unweighted multi-model mean is the best estimate^42–44^. We note that not all reconstructions share the same set of simulation experiments. Uncertainty is calculated as ensemble spread based on bootstrapping with 1000 bootstrap replicates and is expressed as 90% confidence intervals throughout.

### Attribution

Attribution is based on differencing^9^. For CMIP5 and MsTMIP, we assume the difference between two simulations is solely attributable to the relevant factor, e.g., subtracting SG2 from SG3 recovers the effect of time-varying [CO_2_] as both MsTMIP simulations are identical apart from SG3 having dynamic [CO_2_] enabled. Similarly, in CMIP5, subtracting piControl from historicalNat GPP recovers the effect of natural forcings only as both simulations are identical apart from historicalNat including changes in natural forcings–solar irradiance and volcanic aerosols.

For climatic factors (near-surface air temperature, precipitation, and downwelling shortwave radiation) the effect of each is recovered using a machine learning-based emulator of MsTMIP run SG1 (time-varying climate only). Here the random forest algorithm^45^ is trained with SG1 GPP as the target and near-surface air temperature, precipitation, and downwelling shortwave radiation as explanatory variables. The explanatory variables are those used in forcing all offline MsTMIP runs and are identical across all MsTMIP models. Training is done by grid cell at monthly time step for each MsTMIP model individually. For the SG1 emulation (Extended Data Fig. 4) median variance explained, in a least-squares sense based on those observations not used in training (out-of-bag data points), is at least 93% across all models and all grid cells. As the climate space used to force RG1 is a randomized subset of that used to force SG1 (ref. ^33,34^) the random forest algorithm does not have to extrapolate beyond the limits of the training data. As such the emulation of RG1 shows equivalent skill (Extended Data Fig. 4) with median variance explained of 96%. For both emulations, skill across space is highly similar with the lowest skill in the Indonesian tropics and Australia and the highest skill across the extratropical Northern Hemisphere.

The effect of each climatic factor is then calculated by differencing after sequentially enabling downwelling shortwave radiation, precipitation, and then near-surface air temperature in the emulator. As an illustration, the effect of downwelling shortwave radiation is recovered based on the difference between two emulations: the RG1 case (all climate explanatory variables use RG1 climate) subtracted from the emulation where SG1 downwelling shortwave radiation is paired with RG1 precipitation and RG1 near-surface air temperature. While the emulator offers a computationally inexpensive approach to attribute changes in GPP to individual climate drivers it is not process-aware but rather maps changes in climate to instantaneous changes in GPP. As such, the emulator provides only a first-order assessment (interaction terms are excluded) of how each climate driver impacts GPP without having to formally execute additional MsTMIP simulations. It is important to note that climatic effects cannot be precisely attributed to anthropogenic or natural forcings. In MsTMIP prescribed forcing data is based on historical information, i.e., contains a mix of anthropogenic changes as well as natural variability in Earth’s climate system.

In total we attribute changes in GPP from 1901 to 2010 to seven single factors and two factor sets: climate, LULCC, [CO_2_], nitrogen deposition, near-surface air temperature, precipitation, and downwelling shortwave radiation as well as all natural forcings (changes in solar irradiance and volcanic aerosols) vs. all anthropogenic forcings (with emphasis on well-mixed greenhouse gases and LULCC; see Extended Data Table 1). We note that attributed changes at 1901, the start of the analysis period, are not *a priori* zero as anthropogenic impacts on carbon cycling predate 1901.

## Data Availability

MsTMIP Version 1 data are available without restriction through the Oak Ridge National Laboratory’s Distributed Active Archive Center (ORNL DAAC; https://daac.ornl.gov/) at 10.3334/ORNLDAAC/1225. CMIP5 data are available without restriction through the Earth System Grid Federation (ESGF) as hosted at the Lawrence Livermore National Laboratory at https://esgf-node.llnl.gov/projects/esgf-llnl/.
