# Author Correction: Modeling suggests fossil fuel emissions have been driving increased land carbon uptake since the turn of the 20th Century

**DOI:** 10.1038/s41598-020-70342-1

**Published:** 2020-08-10

**Authors:** Christopher R. Schwalm, Deborah N. Huntzinger, Anna M. Michalak, Kevin Schaefer, Joshua B. Fisher, Yuanyuan Fang, Yaxing Wei

**Affiliations:** 1 Woods Hole Research Center, Falmouth, MA 02540 USA; 2 Center for Ecosystem Science and Society, Northern Arizona University, Flagstaff, AZ 86011 USA; 3 School of Earth & Sustainability, Northern Arizona University, Flagstaff, AZ 86011 USA; 4 Department of Global Ecology, Carnegie Institution for Science, Stanford, CA 94305 USA; 5 National Snow and Ice Data Center, Boulder, CO 80309 USA; 6 Jet Propulsion Laboratory, California Institute of Technology, 4800 Oak Grove Dr., Pasadena, CA 91109 USA; 7 Environmental Sciences Division, Oak Ridge National Laboratory, Oak Ridge, Tennessee 37831 USA

Correction to: *Scientific Reports*10.1038/s41598-020-66103-9, published online 03 June 2020

The original version of this Article contained a typographical error in the spelling of the author Deborah N. Huntzinger, which was incorrectly given as Deborah N. Huntinzger. This has now been corrected in the PDF and HTML versions of the Article.

